# The Identification of the Deformation Stage of a Metal Specimen Based on Acoustic Emission Data Analysis

**DOI:** 10.3390/s17040789

**Published:** 2017-04-07

**Authors:** Shenao Zou, Fengying Yan, Guoan Yang, Wei Sun

**Affiliations:** 1 College of Mechanical and Electrical Engineering, Beijing University of Chemical Technology, Beijing 100029, China; zoushenao@163.com; 2 Beijing Key Lab of Membrane Science and Technology, College of Chemical Engineering, Beijing University of Chemical Technology, Beijing 100029, China; yfy2013@163.com

**Keywords:** acoustic emission, metal deformation degree, tensile test, signal energy ratio, empirical mode decomposition

## Abstract

The acoustic emission (AE) signals of metal materials have been widely used to identify the deformation stage of a pressure vessel. In this work, Q235 steel samples with different propagation distances and geometrical structures are stretched to get the corresponding acoustic emission signals. Then the obtained acoustic emission signals are de-noised by empirical mode decomposition (EMD), and then decomposed into two different frequency ranges, i.e., one mainly corresponding to metal deformation and the other mainly corresponding to friction signals. The ratio of signal energy between two frequency ranges is defined as a new acoustic emission characteristic parameter. Differences can be observed at different deformation stages in both magnitude and data distribution range. Compared with other acoustic emission parameters, the proposed parameter is valid in different setups of the propagation medium and the coupled stiffness.

## 1. Introduction

Pressure vessels have been widely used in the process industry. A unexpected vessel leak not only interrupts production in an industrial facility, but also endangers personal safety [1]. Great attention has been paid to monitor and access its working condition in both industry and academia. Most vessel leaks are caused by irreversible deformation of metal material [2]. The deformation of metals can be graded into four stages: elastic stage, yield stage, uniform plastic stage, and necking stage. Different deformation stages correspond to different degrees of damage of the metals. At the elastic stage, a linear relationship between stress and extension is usually observed for most metals and there are no damages for the metals after the external force is removed. The permanent plastic deformation happens at the yield stage. Then obvious plastic deformation is observed, which marks the coming of the uniform plastic stage. It would be very dangerous if no proper measures are applied. An accident happens when the necking stage starts [3,4]. So the deformation degree has been widely used to characterize the condition of pressure vessels while in service [5]. There are different methods available for the detection of metal deformation. For example, ultrasonic detective method is widely used to monitor the slow fatigue crack growth in aluminum and magnesium alloys, where atmospheric moisture is a significant factor which may influence the endurance data [6]; Fiber optic Bragg grating (FOBG) method is an effective way to monitor the true strain on the surface of specimens, but the main problem is to define the mounting of the FOBG sensors on the specimens [7]; A visualization system utilizes a 2D laser displacement sensor to capture the deforming profile of the workpiece during operation, and displays the results in graphical form. This provides engineers and researchers with an intuitive means of analyzing and diagnosing the deformation process during sheet metal forming [8]. In general, they can be approximately classified as acoustic emission (AE) technique, thermal infrared testing method, ultrasonic method, resistance method, etc. The AE technique, as the only non-destructive examination (NDE) method, is able to assess volumetric integrity during a pressure vessel under loading [9,10,11,12,13,14]. The main AE during the deformation of metals is generated from massive dislocation activities in grain boundaries [15]. First dislocation activities take place at the yield stage. Then intensive increasing of dislocation activities is observed and a Luders band is formed eventually. Plastic deformation begins in this stage. At uniform plastic stage, AE activities are observed, too. The decrease of AE counts in this stage is connected with the reduction of dislocation activities. There are no dislocation activities at other two stages [16,17]. Meanwhile, the AE activities in the deformation of metals follow the Kaiser effect, first investigated by Joseph Kaiser, which expressed that little or no AE signals will be recorded before the previous maximum stress level is achieved [18]. For example, if the external force is unloaded at uniform plastic stage, there will be little or no AE signals recorded if external force is introduced again until it reaches the previous uniform plastic stage.

There exists a one to one correspondence between obtained AE signals and dislocation activities in different deformation stages [19]. If the obtained AE signals are processed properly, the deformation degree of metals can be identified correctly. However, the obtained AE signals are always accompanied by frictions between sample and device, and background noises in both the measurement device and the environment [20]. Furthermore, AE signals from the deformation of metals are usually nonlinear and non-stationary [21]. General signal processing methods, such as Fourier Transform, cannot fulfill the requirements of processing the result. A number of studies have been carried out to develop methods for filtering out the noise signals [20,22]. Wavelet transform (WT) and empirical mode decomposition (EMD) have been widely used in those methods [23,24]. Both transforms enable separation of the AE signals into different frequency ranges. Compared with WT, EMD calculation does not involve any convolution, which could save some computational time [25]. However, EMD calculation generates undesirable intrinsic mode functions (IMF) at a low-frequency range, which may cause misinterpretation [26]. On the contrary, it is hard to obtain a satisfactory resolution using WT analysis at a high-frequency range [25]. The extracted AE signals are mainly characterized by AE count, rise-time, amplitude, or energy. In these characteristics parameters, the AE count is used to estimate different deformation stages of metals [27], and the distributions of amplitude or energy of AE signals are also consistent with the internal dislocation movement of metals [28]. It is also reported that the change of characteristics parameters is influenced by the propagation medium, the coupled stiffness, and the AE sensor [29].

Most methods proposed in literature are based on the AE signals received from a measurement device, with no further consideration of the signal source [30]. When AE signals are recorded, it contains the information from its source, propagation medium, coupled stiffness, and AE sensor. They can be expressed by the characteristic or transfer functions of the source, the propagation medium, the coupled stiffness, and the sensor ($H_{s}$, $H_{m}$, $H_{c}$, and $H_{t}$). In frequency domain, the transfer function of the AE signals, $H_{AE}$, is given by the product of the four transfer functions above [31,32]:

(1) $$H_{AE}=H_{s}\cdot H_{m}\cdot H_{c}\cdot H_{t}.$$

$H_{AE}$ is the combination of all these factors. In order to improve analysis results, the feature information of the $H_{s}$ shall be discussed in detail. The $H_{s}$ includes tensile source, friction source, and other environmental sources [33]. At the same time, the frequencies of the three AE sources mentioned above, from certain failure modes, almost remain unchanged [16]. One of the most significant researches on dynamic deformation degree of metals is the frequency characterization of the tensile source and some noise sources during the deformation of metals.

It has been proved that the friction signals do not follow the Kaiser effect, which is different from the AE signals of metal deformation [34,35]. In other words, the total energy of original friction signals remains unchanged during the deformation of metals [35]. The difference of the received friction signals at different degrees of metal deformation results from the propagation medium, the coupled stiffness, and the AE sensor, while these factors affect other signals at the same time. Frequencies of received signals are corresponding to those of their source signals [22], which makes it possible to separate signals from different sources by frequency analysis. If the friction signals could be extracted and used as a baseline, it may help to reduce the influence of the propagation distance, the coupled stiffness, and the sensor. For this purpose, the tensile experiments with different propagation distances and geometry structures are designed and analyzed in Section 2. The rest of this paper is organized in the following manner. In Section 3, the filtering methods are introduced, and a new AE characteristic parameter is proposed. The filtering and deformation degree results and discussion are included in Section 4. The conclusions are drawn in Section 5.

## 2. Experimental

Q235 is the common material of pressure vessels, in addition to the element iron, it contains relatively numerous amounts of carbon, manganese, silicon, sulfur, and phosphorus. Specimens made of Q235 plain carbon steels with two different geometry structures were chosen for tensile test (Table 1) in this work. Five different propagation distances of elastic waves are adjusted by the distance between fracture and the location of sensor.

**Figure 1:**
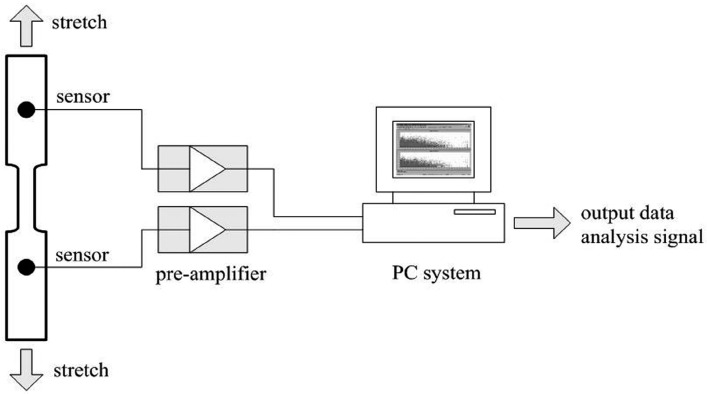
The tensile environment and schematic of testing system.

The measurement of the stress-time curve was obtained by an MTS-810 electro-hydraulic servo-controlled testing machine with a maximum loading of 10 t. AE signals were collected by a multi-channel SAMOS acoustics emission system, which is composed of a PC system, pre-amplifiers and sensors. An R15-ARPHA sensor was selected in this tensile test with a peak frequency of 150 kHz, so that the environmental noises can be eliminated to some extent. The AE sensors were attached firmly to the surface of the substrate by a thin film of Vaseline to ensure maximum ultrasonic signal transmission. The tensile system and acoustic emission system were connected by cables so that the load–time curves and AE characteristics could be recorded simultaneously. Figure 1 shows the tensile environment and schematic of the testing system.

AE data from three channels were recorded for all tests. In this system, AE signals that traveled through tensile specimen were received first, AE signals were then filtered and magnified by an AEwin TM analyzer, and recorded automatically by a computer at the end. 

## 3. Methods

The obtained AE signals contain signals from metal deformation, friction signals, and environmental noise. Only signals from metal deformation and friction signals are of interest in this work. Thus, they should be extracted first. Then a new AE characteristic parameter will be studied with the aim of eliminating the influence of the propagation medium, the coupled stiffness, and the sensor. 

### 3.1. The Filtering Methods

**Figure 2:**
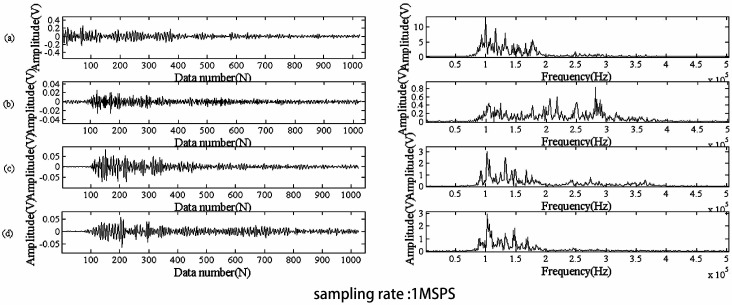
Plots in the time domain (**left**) and the frequency domain (**right**) for obtained acoustic emission signals: (**a**) elastic stage; (**b**) yield stage; (**c**) uniform plastic stage; and (**d**) necking stage.

The analysis of AE signals from the deformation of metals is seriously affected by environmental noise and friction signals. The AE signals during deformation in the time domain and the frequency domain are plotted in Figure 2.

As indicated in the previous section, frequency domain methods are available to filter out noise contents from the original signals. In this work, both friction signals and signals from metal deformation need to be preserved after filtering. Most of them exist in a high-frequency range. Compared with WT, it is easy to obtain a satisfactory resolution using EMD analysis at the high-frequency range [25]. Also, to use WT, an appropriate wavelet base function had to be selected beforehand.

EMD is one of the elements of Hilbert-Huang Transform (HHT) proposed by Norder E. Huang in 1998 [36]. In EMD, the intrinsic oscillatory modes are identified by their characteristic time scales in the signals, and then the signals are decomposed into a collection of IMFs. The IMF, containing different local features of the original signals, can be employed to express the original signals in a complete, adaptive, and orthogonal way. 

The IMF satisfies the following properties: (1) the number of extrema of IMF is equal to, or at most different by one to the number of zero crossings, (2) the mean value between the maximum and the minimum value of the envelope is equal to zero at any point [25]. The IMF satisfying the above conditions is a mono-component signal. Thus, the decomposition method is used with the envelopes composed by the local maxima and minima separately. Once the extrema are ascertained, all the local maxima are connected by a smooth line curve as the upper envelope $x_{\max}(t)$. The lower envelope, named $x_{\min}(t)$, can be obtained by the same procedure. Their mean is designated as $m_{1}(t)$ and the difference between the original signal, X(t) and $m_{1}(t)$ is the first component $h_{1}(t)$, i.e.,

(2) $$m_{1}(t)=\frac{x_{\max}(t)+x_{\min}(t)}{2}$$

(3) $$h_{1}(t)=X(t)-m_{1}(t).$$

The previous calculation will be repeated to change the original signal to $h_{1}(t)$ until the first IMF is obtained as $C_{1}(t)$. The difference between X(t) and $C_{1}(t)$ is the next original signal $r_{1}(t)$, i.e.,

(4) $$r_{1}(t)=X(t)-C_{1}(t)\;r_{2}(t)=r_{1}(t)-C_{2}(t)\;\ldots \;r_{n}(t)=r_{n-1}(t)-C_{n}(t).$$

As $r_{n}(t)$ cannot meet the requirements of the IMF, this also means the end of EMD decomposition, i.e.,

(5) $$X(t)=\sum_{i=1}^{n}C_{i}(t)+r_{n}(t).$$

Flandrin [37] and Wu [38] have done a lot of statistical analysis on the EMD results of fractional Gauss noise and white Gaussian noise, and the distribution characteristics of power spectral density of EMD in different IMF components were obtained. Using this distribution, EMD can be effectively used for signal de-noising.

With repeated sifting, different frequency bands can be separated clearly. The IMFs with the requisite frequency are picked up to recombine the signal without the noise content [17,39].

### 3.2. The Ratio of Signal Energy between Two Frequency Ranges

As discussed in the previous section, the obtained AE signal after de-noising, $H_{AE}$, is considered the combination of the AE signal from metal deformation, $H_{TAE}$, and the AE signal from friction force, $H_{FAE}$. Both of them contain the information from their sources, propagation medium, coupled stiffness, and AE sensor. The influence of the propagation medium, the coupled stiffness and the sensor are equally applied [31,32], as shown in Equation (6)

(6) $$H_{TAE}=H_{ts}\cdot H_{m}\cdot H_{c}\cdot H_{t}\;H_{FAE}=H_{fs}\cdot H_{m}\cdot H_{c}\cdot H_{t}$$

where $H_{ts}$ stands for the AE source from tensile deformation and $H_{fs}$ stands for the AE source from fraction force.

The signals containing both $H_{TAE}$ and $H_{FAE}$ could be obtained by their characteristic frequencies. And the influence of $H_{m}$, $H_{c}$, and $H_{t}$ remains the same. The ratio of overall signal energy at tensile frequency range to overall signal energy at frictional frequency range, is proposed as the AE characteristic parameter, as shown in Equation (7), which will be referred as the ratio of signal energy thereafter.

(7) $$R=\frac{H_{TAE}}{H_{FAE}}=\frac{H_{ts}\cdot H_{m}\cdot H_{c}\cdot H_{t}}{H_{fs}\cdot H_{m}\cdot H_{c}\cdot H_{t}}=\frac{H_{ts}}{H_{fs}}$$

Then the influence of the propagation medium, the coupled stiffness, and the sensor could be cancelled by keeping parameter settings of all the instruments constant. As there is no Kaiser effect in friction signals, the total energy of friction signals shall stay unchanged with the process of metal deformation. There shall be a one-to-one correspondence between $H_{ts}$ and R along the progress of metal deformation, i.e., the deformation degree of metals can be reflected by the value of R. 

Once the above discussion is validated by experiment, there shall be a series of *R* values corresponding to degrees of metal deformation, therefore, the degree of metal deformation can be identified by the analysis of AE collected on site. More optimistically, the idea can be extended to the monitoring of pressure vessels with load.

## 4. Results and Discussion

**Figure 3:**
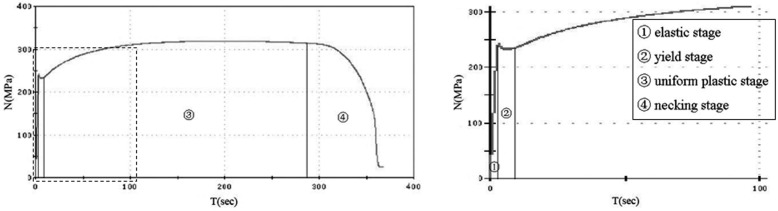
The stress-time curve.

The AE signals used in this work are introduced at Table 1. In every tensile test, tens of thousands of AE events are collected. All AE events can be divided into four parts according to the time of the stress-time curve shown in Figure 3, which is synchronized with an acoustic emission system. The right part in Figure 3 is local detail of the selected part on the left figure. Signals are de-noised first and then the characteristic parameter is extracted based on the discussion in Section 3. 

### 4.1. De-Noising by EMD 

**Figure 4:**
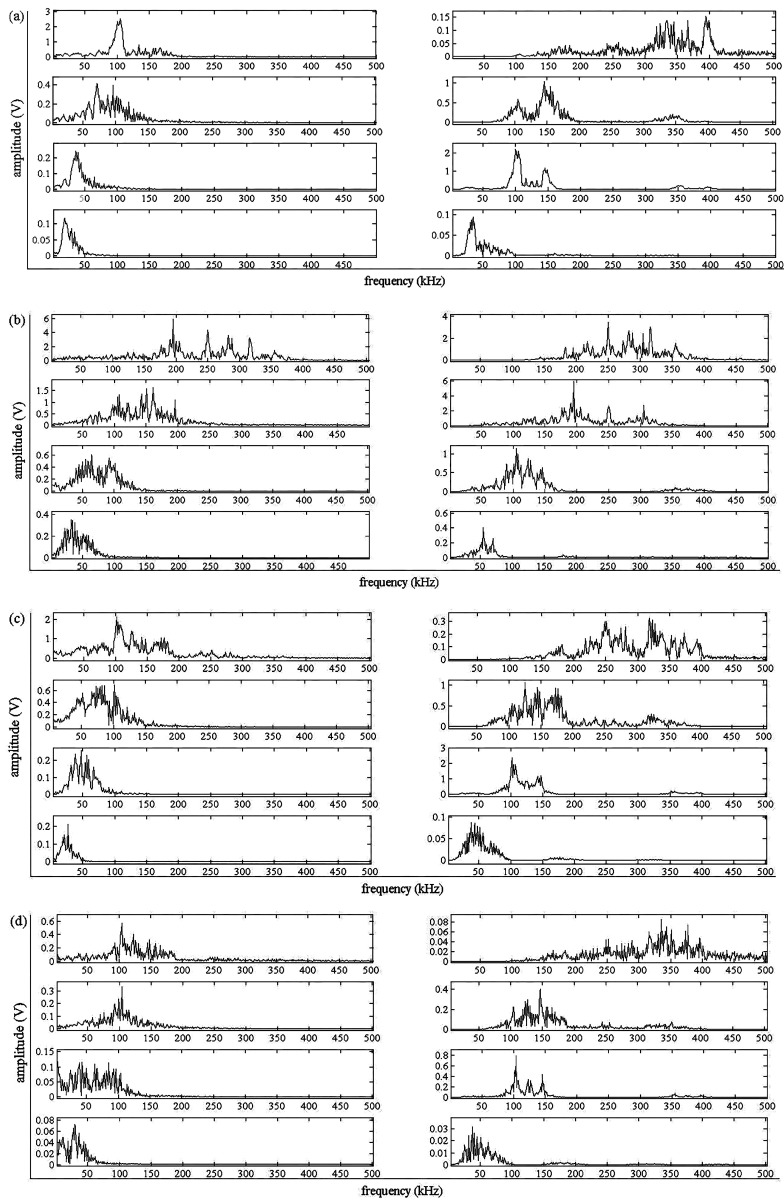
De-noising results by empirical mode decomposition (**left**) and wavelet transform (**right**) during tensile tests: (**a**) elastic stage; (**b**) yield stage; (**c**) uniform plastic stage; and (**d**) necking stage.

The goal of de-noising in this work is to remove the components with frequencies less than 90 kHz. Both WT and EMD methods are employed for this purpose, and their results are compared in Figure 4.

Figure 4 shows the de-noising results of AE signals during four the deformation stages in the tensile process. The left part of the dash line is decomposed by EMD and the right part is processed by WT. It can be seen that the distribution of frequency components analyzed by EMD are notably concentrated. Based on the signal analysis in the four deformation stages, EMD shows an adaptive capacity without any false signals in a high frequency range, where the AE signal associated with deformation and friction signal resides. Therefore, in the following work, the EMD is employed to extract the signals with frequencies higher than 90 kHz.

### 4.2. Ratio of Signal Energy Analysis and Feature Extraction

**Figure 5:**
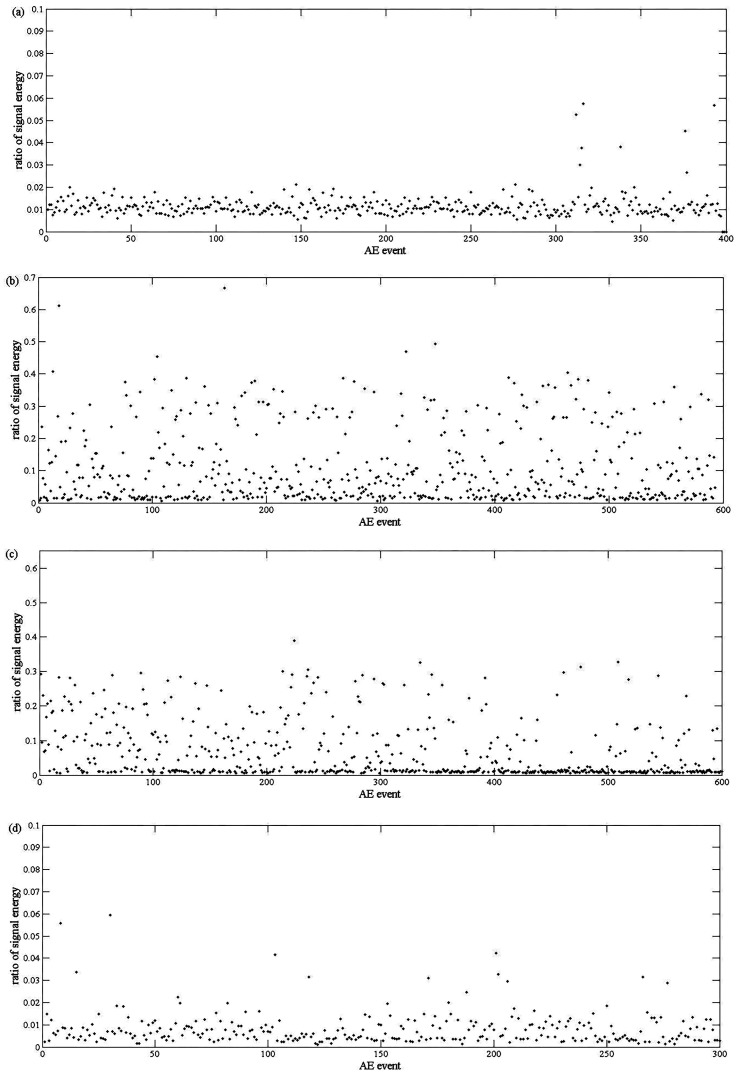
Ratio of the signal energy between two frequency ranges: (**a**) elastic stage; (**b**) yield stage; (**c**) uniform plastic stage; and (**d**) necking stage.

**Figure 6:**
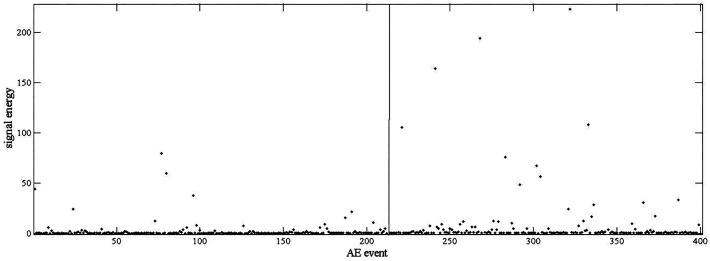
Signal energy distribution of the elastic stage (**left**) and the yield stage (**right**).

For more accurate analysis, the de-noised signals are recombined by IMFs with frequencies higher than 100 kHz, and then decomposed into two different frequency ranges. The ratio of signal energy between the two frequency ranges is calculated. All four stages of scheme 1, at Table 1, are analyzed and depicted in Figure 5 respectively. As a comparison, Figure 6 shows the signal energy distribution of the elastic stage and the yield stage in frequency domain.

The investigation on low carbon steel showed that the major source of AE in a tensile process is associated with dislocation activity in the grain boundaries and the intensive motion of slip bands [16,17], as introduced in Section 1. In the yield stage, new dislocations are generated and slip bands are eventually spread. Most of the ratios of signal energy keep a larger value as shown in Figure 5b. In the uniform plastic stage, the decrease of AE counts results from the reduction of dislocation activities. The ratios of signal energy in Figure 5c are smaller. In the elastic stage and the necking stage, no dislocation activity exists. The ratios of signal energy in Figure 5a,d remain below 0.1, however, the elastic stage and the necking stage can still be determined, since the device deformation have to go through the elastic stage and uniform plastic stage before the emergence of the necking stage, and these two stages are easy to determine as showed in Figure 5b,c. In conclusion, the ratio of signal energy analysis is in accordance with theoretical analysis of a tensile process. In fact, it is only needed to determine between the elastic stage and the yield stage, because the monitored device is no longer fit to serve anymore, once the yield stage of deformation is reached. These two stages are easy to determine by comparing Figure 5a,b. However, in Figure 6, there is no obvious difference between signal energy at the elastic stage and the yield stage. As a result, the ratio of signal energy is more appropriate in this condition.

**Figure 7:**
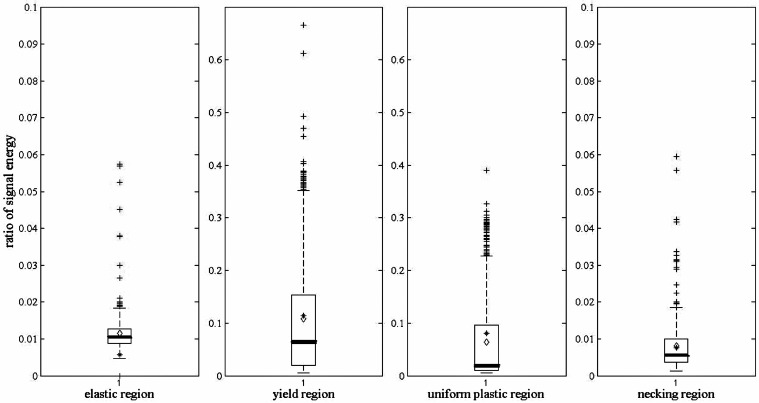
Ratio of signal energy diagram of the four deformation stages.

All AE events in the four deformation stages are statistically analyzed by the ratio of signal energy and then illustrated by box-plot as shown in Figure 7, where the highest point is the maximum, the lowest point is the minimum. The bottom and top points of the box are the 25th and the 75th percentile, while the bold line inside the box is the median. In order to summarize the ratio of signal energy distribution in different stages clearly, the mean and standard deviation are shown in Table 2.

Differences can be observed on two major aspects, the ratio value magnitude and the data distribution range, which are consistent with the above theoretical analysis during a tensile process.

**Figure 8:**
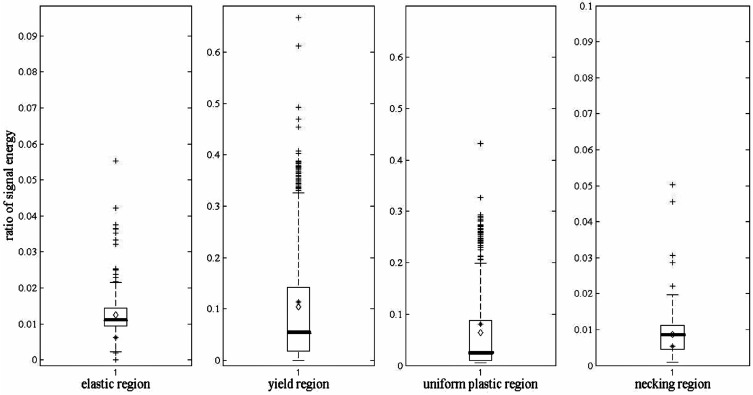
Ratio of signal energy diagram of the four deformation stages.

In order to further test this method in a different propagation path and coupled stiffness, the other AE signals from the tensile test are analyzed. One of them is with a different vibration transmitting distance. As mentioned previously, Figure 7 shows the ratio of signal energy diagram of scheme 1, at Table 1. Accordingly, the ratio of signal energy diagram of scheme 5, at Table 1, is shown in Figure 8. The mean and standard deviation of the ratio with other different vibration transmitting distances are also listed in Table 2.

**Figure 9:**
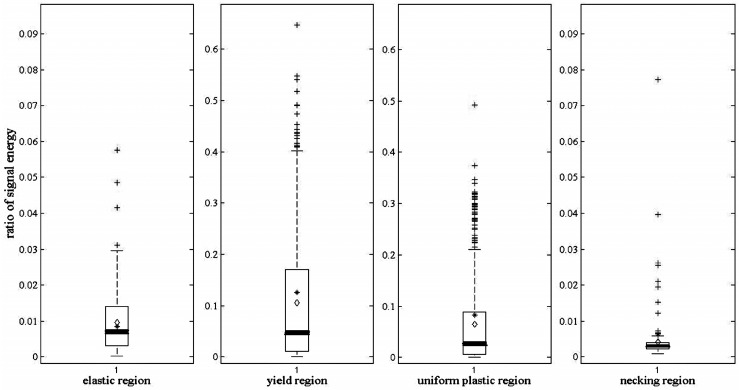
Ratio of signal energy diagram in four deformation stages.

The specimen geometry in Figure 7 and Figure 8 is 8 mm in width, 5 mm in thickness, and 88 mm in length, which is introduced at Table 1. Correspondingly, for the specimen in Figure 9, its geometry is 12 mm in width, 3 mm in thickness, and 60 mm in length, which is named as scheme 6, at Table 1.

The mean and standard deviation of each scheme at different stages of tensile test are also illustrated in Table 2.

The results in Figure 8 and Figure 9 are similar with those in Figure 7. According to Table 2, the ratio of signal energy between two frequency ranges remains nearly the same in all six different schemes regardless of their geometries and distances from sensor location. It affirms that the ratio can be used as an indicator to identify the deformation degree of certain metals.

## 5. Conclusions

In this work, a new AE characteristic parameter, a ratio of signal energy at tensile frequency range to signal energy at frictional frequency range, is proposed. Since the AE signal associated with metal deformation is affected by the degree of coupling, the propagation distances, and the geometry structures, signal energy is not appropriate to determine the deformation degree of the metal. However, the ratio of signal energy can be used to determine the deformation degree of metals regardless of the devices’ geometries and distances from the sensor location as mentioned above, which is based on the assumption that the produced friction signals remain unchanged during the process of tension. In order to extract signals from metal deformation and friction signals, an EMD calculation was employed in this study. When the data volume is large enough and the mechanical testing machine is precise enough, the condition monitoring and assessment of pressure vessels can be achieved based on AE signal analysis. 

## Figures and Tables

**Table 1:** Geometry structure of tensile specimen and the distance between fracture and the location of the sensor.

Scheme	Geometry Structure			Distance between Fracture and the Location of Sensor (mm)
	Width (mm)	Thickness (mm)	Length (mm)	
1	8	5	88	115
2	8	5	88	110
3	8	5	88	105
4	8	5	88	100
5	8	5	88	90
6	12	3	60	90

**Table 2:** The mean and standard deviation of the ratio of signal energy for different AE signals at the different deformation stages.

Eigenvalue	Scheme	Elastic Stage (%)	Yield Stage (%)	Uniform Plastic Stage (%)	Necking Stage (%)
mean	1	0.0125	0.1082	0.0645	0.0081
	2	0.0101	0.1256	0.0736	0.0094
	3	0.0139	0.1090	0.0667	0.0083
	4	0.0103	0.1200	0.0639	0.0086
	5	0.0124	0.104	0.0639	0.0086
	6	0.0096	0.1050	0.0643	0.0081
standard deviation	1	0.0057	0.1145	0.0808	0.0075
	2	0.0060	0.1209	0.0830	0.0054
	3	0.0052	0.1107	0.0774	0.0067
	4	0.0068	0.1222	0.0811	0.0055
	5	0.0062	0.1145	0.0811	0.0056
	6	0.0084	0.1255	0.0830	0.0065
